# Reactive Oxygen Species *via* Redox Signaling to PI3K/AKT Pathway Contribute to the Malignant Growth of 4-Hydroxy Estradiol-Transformed Mammary Epithelial Cells

**DOI:** 10.1371/journal.pone.0054206

**Published:** 2013-02-21

**Authors:** Victor O. Okoh, Quentin Felty, Jai Parkash, Robert Poppiti, Deodutta Roy

**Affiliations:** 1 Department of Environmental and Occupational Health, Florida International University, Miami, Florida, United States of America; 2 Department of Pathology, Florida International University, Miami, Florida, United States of America; Catholic University Medical School, Italy

## Abstract

The purpose of this study was to investigate the effects of 17-β-estradiol (E2)-induced reactive oxygen species (ROS) on the induction of mammary tumorigenesis. We found that ROS-induced by repeated exposures to 4-hydroxy-estradiol (4-OH-E2), a predominant catechol metabolite of E2, caused transformation of normal human mammary epithelial MCF-10A cells with malignant growth in nude mice. This was evident from inhibition of estrogen-induced breast tumor formation in the xenograft model by both overexpression of catalase as well as by co-treatment with Ebselen. To understand how 4-OH-E2 induces this malignant phenotype through ROS, we investigated the effects of 4-OH-E2 on redox-sensitive signal transduction pathways. During the malignant transformation process we observed that 4-OH-E2 treatment increased AKT phosphorylation through PI3K activation. The PI3K-mediated phosphorylation of AKT in 4-OH-E2-treated cells was inhibited by ROS modifiers as well as by silencing of AKT expression. RNA interference of AKT markedly inhibited 4-OH-E2-induced *in vitro* tumor formation. The expression of cell cycle genes, cdc2, PRC1 and PCNA and one of transcription factors that control the expression of these genes – nuclear respiratory factor-1 (NRF-1) was significantly up-regulated during the 4-OH-E2-mediated malignant transformation process. The increased expression of these genes was inhibited by ROS modifiers as well as by silencing of AKT expression. These results indicate that 4-OH-E2-induced cell transformation may be mediated, in part, through redox-sensitive AKT signal transduction pathways by up-regulating the expression of cell cycle genes cdc2, PRC1 and PCNA, and the transcription factor – NRF-1. In summary, our study has demonstrated that: (i) 4-OH-E2 is one of the main estrogen metabolites that induce mammary tumorigenesis and (ii) ROS-mediated signaling leading to the activation of PI3K/AKT pathway plays an important role in the generation of 4-OH-E2-induced malignant phenotype of breast epithelial cells. In conclusion, ROS are important signaling molecules in the development of estrogen-induced malignant breast lesions.

## Introduction

Elevated lifetime estrogen exposure is a well-known major risk factor for breast cancer. A large body of epidemiological and experimental evidence points to a role for estrogen in the etiology of human breast cancer [1]–[9]. In experimental models, estrogens are complete breast carcinogens, as they are capable of initiating and triggering growth and selection to generate palpable malignancy [8]–[14]. However, the signaling mechanisms by which estrogen contributes in the initiation of breast cancer remain the subject of a long-standing controversy. This is due, in part, to the inability to resolve whether estrogen or estrogen metabolites are procarcinogenic. 17β-estradiol (E2) is metabolized to 2- and 4-hydroxy-estradiols by cytochrome p450s. We have previously shown that E2-induced renal tumor formation is decreased in animals exposed to inhibitors of estrogen metabolism or to hormonally potent estrogens undergoing reduced metabolic conversion to catechol metabolites compared to E2 [10]–[12], [15]. The research laboratory of Dr. Jose Russo has shown that E2 or 4-OH-E2 transform normal ERα negative breast epithelial MCF-10F cells [16]–[20] to neoplastic cells. 17β-estradiol-induced transformed MCF10F cells form tumors in SCID mice. 4-OH-E2 is twice as capable of producing anchorage-independent growth in MCF10F cells when compared to E2 [18], [20]. In contrast, neither 2-OH-E2 nor 2-OH-E1 are carcinogenic *in vitro* or *in vivo* [15]. Similarly, 17 beta-estradiol and equilenin catechol metabolite, 4-hydroxyequilenin treatment to normal ERα negative breast epithelial MCF-10A cells induced anchorage independent growth in these cells or foci formation [21]–[23]. Recently, Parks et al (2009) demonstrated that MCF10A cells exposed to 4-OHE2 showed anchorage-independent growth [24]. However, these studies in MCF-10A cells failed to show *in vivo* tumorigenicity, invasiveness or display other salient neoplastic properties after estrogen treatment. In the present study, we have conducted comprehensive analyses to show that repeated exposures of 4-OH-E2 to MCF-10A produced neoplastic transformation *in vitro* and transformed cells were found to be tumorigenic *in vivo*.

Induction of estrogen receptor (ER) upon estrogen exposure is not sufficient for the development of breast cancer. Recent studies indicate that mammary tumors can develop in the absence of a functional ERα [25]. Although tamoxifen and other antiestrogens are thought to prevent cancer through their actions at the ER, other mechanisms cannot be ruled out as these compounds also block metabolism and redox cycling of estrogen and are free radical scavengers [26]. 4-OH-E2 induces an estrogenic response in the uterus of ERα null mice, and this response is not inhibited by the antiestrogen ICI182780 [27]. These findings suggest that estrogen-dependent growth of cells is regulated not only by nuclear ER-mediated genomic signaling pathways, but also by non-ER pathway(s). We believe that genomic and non-genomic actions of estrogen produce complementary effects that are required for cellular transformation. Physiologically achievable concentrations of estrogen or estrogen metabolites directly acting on mitochondria of mammary epithelial or immune cells generate reactive oxygen species (ROS) [28]. We previously showed that 17-β-estradiol (E2)-induced DNA synthesis in MCF-7 breast cancer cells depends on mitochondrial oxidant signaling [29]. In this study, we have extended our efforts on understanding how an E2 metabolite, 4-OH-E2 produces malignant phenotype through ROS signaling. We investigated whether the susceptibility of normal breast epithelial MCF-10A cells to neoplastic transformation by estrogens depends on ROS-mediated redox signaling. We present here for the first time that oxidants induced by E2 and 4-OH-E2, but not 2-OH-E2 exposures mediated *in vitro* transformation of MCF-10A cells. 4-OH-E2 transformed cells are not only tumorigenic in mice but also display invasive properties as well as proliferation independent of growth factors. Co-treatments of 4-OH-E2 transformed cells with biological or chemical ROS scavengers, or silencing of AKT1 prevented tumorigenic conversion of MCF-10A cells. It appears that oxidant-mediated activation of redox sensitive PI3K/AKT signaling may be involved in the tumorigenic conversion of normal breast epithelial cells by estrogen.

## Materials and Methods

### Ethics Statement

All experimental procedures for the use of animals were approved by the institutional animal care and use committee (IACUC) at the Florida International University (protocol #09–034), and all of the experiments were conducted in accordance with the Guide for the Care and Use of Laboratory Animals published by the US National Institutes of Health.

### Chemicals and Reagents

17β-Estradiol (E2), 2-hydroxyestradiol (2-OH-E2), 4-hydroxyestradiol (4-OH-E2), Ebselen, N-acetyl-cysteine (NAC), and Dimethylsulfoxide (DMSO) were all purchased from Sigma (St Louis, MO, USA). All antibodies; PI3K (p110), phospho PI3K (p85), phospho-AKT (ser 473) and total AKT antibodies were purchased from Cell Signaling Technology Inc. (Boston, MA). All tissue cultures reagents were purchased from Invitrogen Corporation (CA) unless otherwise specified.

### Culture of MCF-10A cells and Adenovirus gene transfer

Human mammary epithelial cells (MCF-10A) were obtained from American Type Culture Collection (ATCC) and were routinely cultured in phenol red-free DMEM-F12 media (1:1) supplemented with 5% horse serum, hydrocortisone (0.5 μg/ml), insulin (10 μg/ml), epidermal growth factor (20 ng/ml), 100 ng/ml cholera toxin and penicillin-streptomycin (100 μg/ml each) and incubated at 37°C in a humidified atmosphere containing 5% CO_2_. The cell culture media, serum, antibiotics, and growth supplements except cholera toxin (Calbiochem, La Jolla, CA) were purchased from Invitrogen Corp, CA. For experimental purposes, culture media were changed to starvation media (serum free media + antibiotics) and allowed to incubate for 48 hrs prior to commencement of most experiments, unless otherwise indicated. Serum deprivation synchronizes cells in the G_0_/G_1_ phase of the cell cycle.

The Adenovirus-CMV (empty vector), Adenovirus-MnSOD (AdCMVMnSOD), and Adenovirus-Catalase (AdCMVCat) constructs were purchased from ViraQuest, Inc. (North Liberty, IA, USA). The adenovirus constructs used were replication-defective, E1- and E3-deleted recombinant adenovirus [30]. Inserted into the E1 region of the adenovirus genome was either the human MnSOD or catalase gene, both of which are driven by a cytomegalovirus promoter. Cells were seeded in plates at 15%–70% confluence. The following day, cells were infected with adenoviruses over-expressing MnSOD or catalase or vector at 100 MOI in serum free media. Control cells were treated with 100 MOI of the adenovirus-CMV construct. This viral load was determined to achieve greater than 50% growth arrests of MCF-10A cells without significant cell death for the duration of the experiment. Infected cells were cultured for 48 hrs after which cells were used for experiments.

### Akt_1_ RNAi transfections

Pre-designed and verified human shRNA for Akt_1_ and corresponding null vectors were purchased from OriGene (OriGene Technologies, Inc. Rockville, MD). Transfections of cells were carried out in a sub-confluent cell population using FuGENE 6 (Roche) transfection reagents according to the manufacturer's protocol. Briefly, MCF10A cells were seeded in 6 well plates with growth factor supplemented media (SM) overnight. Post seeding, cells were transfected with 2 µl of Fugene-6 (Roche) preincubated for 20 min at room temperature with 0.5 µg plasmid RNAi or its null controls (sham). Forty eight hours post transfection, media were changed to serum-free media and incubated for an additional 48 hrs, after which cells were used for various experiments. Transfection efficiencies ranged between 60–80% as quantified by decreased protein expression levels.

### Cell viability assay

CellTiter-Fluor™ Cell Viability kit was purchased from the Promega Corporation and used according to manufacturer's instructions. Briefly, cells were seeded in 96 well plates at a density of 1.0×10^4^ cells/well, serum starved for 48 hrs and treated with estrogens or ROS modifiers. At the end of treatment procedure, substrate reagents (GF-AFC) were mixed with substrate buffer and dispensed into wells. This assay measures protease activity in live cells as opposed to MTT or MTS assay kits that measure formation of formazon crystals by mitochondrial enzymes. Plates were read on a fluorescence plate reader at 380–400 nm excitation and 505 nm emission and data is expressed as mean of three experiments +/− SD.

### Cell transformation

The cell transformation was carried out by a modified protocol of Dr. Jose Russo's group [18]. Briefly, MCF-10A cells were seeded at 30% density in a 10 cm dish. After 24 hrs of seeding, media were replaced with stavation media and allowed to culture for 48 hrs, and then cells were subjected to two treatment cycles with E2 or its catechol metabolites. A treatment cycle includes a 48 hr starvation period, 48 hr treatment period (100 ng/ml of either E2, 2-OHE2, and 4-OHE2), and 48 hr recovery period in growth media containing 10% horse serum (HS) and no growth supplements. At the end of two treatment cycles, cells that would be used for immunoprecipitation and Western blot analysis were treated for an additional 30 mins with estrogens, lysed with RIPA buffer, immunoprecipitated and processed for western analysis. For anchorage independent growth assay 5000 cells/well were used for colony formation assays in soft agar.

### Anchorage independent growth

Anchorage independent growth, an indicator of neoplastic transformation of cells, was assessed as previously described by Zhang et al [31]. Briefly, base support agar were made fresh by diluting 1.0% molten agarose mixed with 1∶1 2x culture media (2x DMEM/F12 media, 20% HS, 2x Penicillin- Streptomycin and 200 pg/ml estrogens) to a final 0.5%. Molten agar was left at 42°C in a water bath until dispensed at 200 ul/well in 48 well plates, then allowed to solidify for 4 hrs at room temperature. Top agarose overlay was made fresh by mixing 0.7% molten agarose with 2x culture media containing 5000 cells/well, and then gently overlaid over base agar. Cells were incubated for a minimum of 21 days in a 37°C incubator with 5% CO_2_. Cells were fed every week with top agar layer and colony formation was assumed when cell masses were 100 micron or greater as measured on a Nikon TE2000U inverted microscope (Nikon Corp., USA) with Metamorph software (Universal Imaging, USA). Images were acquired by using an Olympus C-5060 digital camera attached to the Nikon TE2000U inverted microscope with a 4x objective. Four wells were enumerated for each group and data expressed as mean of five wells +/− SD.

### Invasion and ductulogenic assays

In order to determine invasiveness of transformed cells, several colonies were aseptically picked, dissociated with trypsin and cultured for 20–30 passages in a low growth factor media (DMEM F12, 5% FCS, 4 ng/ml EGF, 2.0 ng/ml insulin, 100 ng/ml hydrocortisone, 1x Penicillin-Streptomycin) and eventually DMEM/F12 with 5% HS media, and 1x Penicillin-Streptomycin. Cells (5.0×10^3^ cells/ml) for invasion assays were seeded over 8 μm pore transwell filter insert (Transwell, Coastar Cambridge, MA) precoated with Matrigel (Collaborative Research, Bedford, MA). Chemo-attractants used were reduced growth factor supplemented media or media with 10% FBS media positive control cells MDA MB 231. Matrix invasion was allowed for 16 hrs at 37°C in a CO_2_ incubator. The non-invaded cells inside chambers were wiped off with a cotton swab, and the filters were fixed, stained by Diff Quick (Sigma, St. Louis, MO), cut out and mounted onto glass slides. The total number of cells that crossed the membrane were counted under a light microscope, enumerated and expressed as fold increase compared to parent cell line. The experiments were repeated five times and results are expressed as the mean ± SD.

For ductulogenesis, 1.0×10^3^ cells/ml of transformed cells (p121 and screened from 3x Matrigel), MDA MB 231 and parental MCF-10A cells were mixed with collagen (Collagen Co., Palo Alto, CA, USA) and seeded in chamber slides precoated also with collagen. Cells were incubated for three weeks with bi-weekly feeding with 5% HS media. To confirm spheroid formation from collagen matrix, we diluted HuBiogel, a human matrix mimetic (VIVO Biosciences Inc.), 1∶3 with media and coated 0.22 micron pore transwell filter inserts for 6 hrs at 37°C incubator. Cells (1.0×10^3^) were seeded into each insert and chemoattractant media (DMEM/F12, 5% HS, 1x Penicillin-Streptomycin) were added at bottom of insert. Cells were cultured for 14 days with media changed twice weekly. Images were acquired with an Olympus C-5060 digital camera under an inverted microscope with a 4x objective as described above.

### Chemical antioxidant treatments

The treatment procedure for Ebselen (a glutathione peroxidase mimetic which also removes both H_2_O_2_ and peroxynitrite) or NAC (a precursor of glutathione and scavenger of ROS) [32], [33] varies according to the experiments design. For all experiments, 40 μM Ebselen and 1.0 mM NAC were used for cell treatments. For example, in DCF assays, antioxidants were pre-loaded onto cells for 2–4 hrs before ROS measurement commences. For BrdU assays, cells were cultured with the chemical antioxidants throughout the experimental procedure. For transformation regimen, antioxidants were applied to cells each time cells were treated with estrogens. For anchorage independent growth assays, antioxidants were added to soft agar matrix media and during weekly feeding of colonies.

### Measurement of reactive oxygen species (ROS)

Cellular ROS were measured on a 96 well plate reader and confocal fluorescence microscopy as previously described by Felty et al [28]. Briefly MCF-10A cells were seeded at a concentration of 1.0×10^4^ cells per well in black 96-well flat bottom plates (Thermo Fisher Scientific Inc. USA) and allowed to adhere overnight. Post seeding, cells were serum starved for 48 hrs after which they were pretreated for 4 hrs with chemical antioxidants Ebselen or NAC (Sigma USA) diluted in Hank's balanced salt solution (HBSS) followed by incubation with 10 µM 2′,7′-dichlorofluorescin diacetate (DCFH-DA) (Invitrogen Corp) for 20 min. Cells were rinsed with HBSS followed by various estrogen treatments as described in the figure legends. DCFH-DA is a non-fluorescent cell-permeable compound, which is acted upon by endogenous esterase that removes the acetate groups generating DCFH. In the presence of intracellular ROS, DCFH is rapidly oxidized to the highly fluorescent 2′,7′-dichlorofluorescein (DCF). The oxidative products were measured with a Tecan Genios 96 well microplate reader using 485 and 535 nm as excitation and emission filters respectively or fluorescence images were acquired on a Nikon TE2000U inverted fluorescence microscope equipped with a Nikon D-Eclipse C1 laser scanning confocal microscope system (Nikon Corp., USA). The built-in Nikon EZ-C1 software was used for confocal image acquisition and analyses. DCFH-DA stock solutions were diluted at a 1∶1 ratio with Pluronic F-127 (20% w/v). Data are expressed as mean of three experiments +/− SD.

### Immunoprecipitation and Western Blot Analysis

After the respective treatments, cells were rinsed twice with ice cold phosphate buffered saline (PBS), harvested with lysis buffer (150 mM NaCl, 0.5% deoxycholate, 0.1% Nonidet P-40, 0.1% SDS, 50 mM Tris) containing protease and phosphatase inhibitors (Roche). Samples were diluted to 500 µg of protein in 1 ml of lysis buffer, and pre-cleared for 1 hr at 4°C with 10 µl of 1∶1 slurry of protein A-agarose beads (Invitrogen Corp) in lysis buffer. After a brief centrifugation to remove pre-cleared beads, 2 µg of desired capture antibodies were added to each supernatant and incubated on a rocking platform at 4°C overnight and captured proteins were precipitated with 40 µl of protein A-agarose beads for 2 hrs. The beads were washed five times with lysis buffer and resuspended in 40 μL sample loading buffer, subjected to electrophoresis and electro-blotted onto a PVDF nylon membrane. Primary antibodies used for Western blots were diluted 1∶1000 in phosphate buffered saline Tween-20**,** PBST and horseradish peroxidase-conjugated secondary antibodies were diluted 1∶50,000 in PBST. Blots were treated with ECL reagents (Amersham Biotech), and proteins were detected by autoradiography. Band intensity was quantified with Bio-Rad Gel Doc Imaging System.

### Immunofluorescence labeling

MCF-10A cells were seeded and treated in chamber slides as indicated in legends to the figures. Post treatment, cells were fixed with ice cold methanol for 15 mins, and permeabilized with 0.5% Triton X-100 for 30 minutes. Cells were blocked with 1% normal goat sera for 1 hr after which they were probed with antibodies diluted 1∶500 for AKT and 1∶500 for phospho AKT. Alexa Fluor labeled secondary antibody directed against AKT antibody was diluted 1∶1000. The confocal fluorescence images were scanned on a Nikon TE2000U inverted fluorescence microscope equipped with a Nikon D-Eclipse C1 laser scanning confocal microscope system (Nikon Corp., USA). The z-series scanning was done at every 1 μm up to a z-depth of 10 μm by using a Nikon 40 x 1.30 NA DIC H/N2 Plan Fluor oil immersion objective. The built-in Nikon EZ-C1 software was used for confocal image acquisition and analyses.

#### Real-Time PCR analysis

The RNA templates (500 ng) were reverse transcribed into cDNA using reverse transcription reagents with random hexamer primers (Applied Biosystems, Foster City, CA, USA). The cDNA was then used as template for real-time PCR with gene specific primers. The TaqMan primers and probe recognizing PCNA, NRF-1, PRC1, CDC2 and 18S were used in this study. Quantitative gene expression analysis was performed by TaqMan-based QRT−PCR on ABI 7700 (PE Applied Biosystems, Foster City, CA, USA). The fold change in gene expression was calculated using the {Delta} Ct method with 18S rRNA as the internal control.

### *In vivo* xenograft growth and histopathology

All protocols involving mice were evaluated and approved by Florida International University's (FIU) Institutional Animal Care and Use Committee and performed under veterinary supervision. Transformed cells (p156) and a corresponding WT control (p178) used for xenograft assay were first screened 3x on Matrigel invasion assay for aggressive phenotype enrichment. Cells that had invaded the matrix at the end of 72 hrs and formed a monolayer at bottom well were cultured and re-verified on colony assay for anchorage independent growth. Upon verification, cells were suspended in 5 mg/ml Matrigel such that each injection of 100 μl bolus contained 5×10^6^ cells. Cell suspensions were injected subcutaneously into 6 week old NCr homozygous nude mice (NCI, Frederick, MD). Tumor growth was monitored by palpation, and the onset when tumors were detectable was noted. Tumor size was measured with calipers, and tumor volume was calculated assuming the shape as ellipsoid. Tumors were removed 33 days post inoculation, weighted and fixed in 10% neutral-buffered formalin for immunohistochemical analysis.

### Histopathology and Ki67 immunocytochemical analyses

Tissues fixed in formalin were embedded in paraffin, cut at 5 µm thickness, mounted on positively charged glass slides, and stained with hematoxylin and eosin for histopathological analysis. For Ki67 fluorescence immunocytochemical analysis, tissue sections were deparaffinized, rehydrated and immuno-labeled as follows. Antigen retrieval was done by first boiling antigen retrieval buffer (10 mM Sodium citrate, 0.05% Tween 20, pH 6.0) in a microwave. Slides were then placed inside the hot buffer for 30 min. Tissue sections were then incubated in diluted normal blocking serum for 20 min. Excess serum was blotted from the slides and the sections were incubated with anti-human Ki67 antibody (DakoCytomation Colorado Inc., Fort Collins, CO, USA). After incubation for 3 hrs, sections were washed in buffer and incubated in Alexa Fluor conjugated secondary antibody for 1 hr. The confocal fluorescence images were scanned on a Nikon TE2000U inverted fluorescence microscope equipped with a Nikon D-Eclipse C1 laser scanning confocal microscope system as described above (Nikon Corp., USA).

## Results

### Exposure of MCF-10A cells to 17β-estradiol (E2) and its metabolites produces a rapid increase in ROS levels

**Figure 1:**
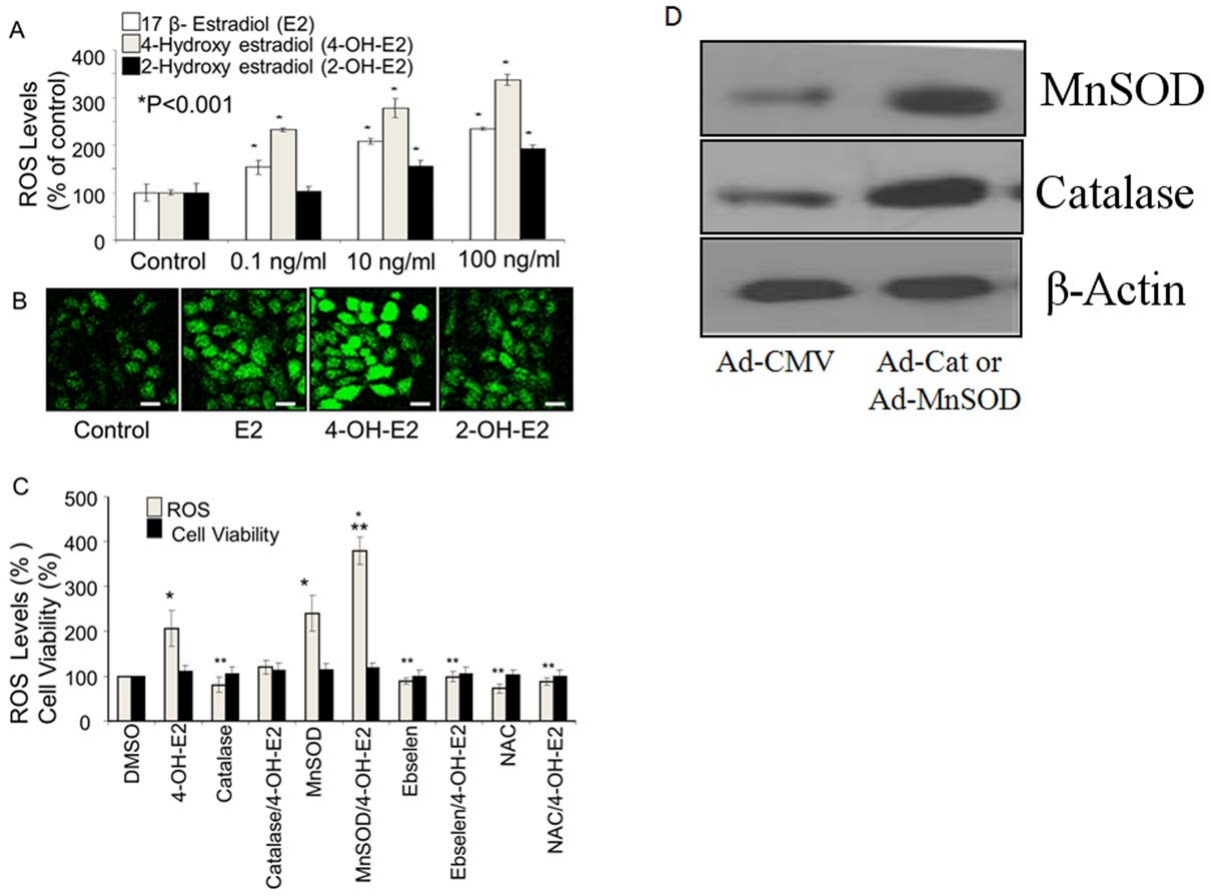
Exposure of MCF-10A cells to 7 β-estradiol (E2) and its hydroxy metabolites produces a rapid increase in ROS levels. ROS production was measured by 10 uM H2DCFDA oxidation on a 96 well plate reader (A&C) and confocal microscopy (B) as previously described by us (28) are shown. **A.** Briefly, H2DCFDA-labeled MCF-10A cells were plated at 3×105 per well of a 96-well plate, and the fluorescence signal obtained was recorded for 5 min. Data represented as mean + S.D. of six independent experiments showing the production of ROS by stimulation of MCF-10A cells with 0.1, 10 and 100 ng/ml of E2 and its hydroxy metabolites, 2-hydroxyestradiol (2-OH-E2) and 4-hydroxyestradiol (4-OH-E2). DMSO (vehicle) was used as a control. Scale bars 20 μm. **B.** Representative confocal images of DCF-DA-loaded MCF-10A cells show that E2 and its hydroxy metabolites trigger release of ROS as detected by a significant increase of DCF-DA fluorescence. **C.** Inhibition of estrogen-induced ROS production by overexpression of catalase or treatment with Ebselen (40 uM) or NAC (1.0 mM). Antioxidants were pre-loaded onto cells for 2–4 hrs before ROS measurement commences. **D.** For overexpression of ROS detoxifying enzymes, cells were infected with adenoviral vectors encoding catalase (Ad-catalase) or MnSOD (Ad-MnSOD) or empty vector (Ad-CMV) as a control at 100 MOI in serum free media. Cells were infected with Ad-catalase or MnSOD of 100 MOI for 48 h. Overexpression of catalase or MnSOD was confirmed by Western blot analysis using antibodies against catalase or MnSOD. β-Actin was processed in parallel as an internal control for protein loading.The cellular protease activities were measured to rule out the possibility that the differential ROS levels were not as a result of differential cell densities or viability. Data represented as mean + S.D. of six independent experiments showing inhibition of estrogen-induced ROS generation by ROS modifiers. DMSO (vehicle) was used as a control. *P<0.05, significantly different from control. **P<0.05 indicates significantly different from 4-OHE2, (P<0.05).

Before carrying out cell transformation, we characterized normal human mammary epithelial cells for their ability to produce ROS in response to 17 beta-estradiol (E2) exposure. These cells respond to E2 in terms of producing ROS very similar to breast cancer cells. ROS production by E2 and its metabolites, 2-OH-E2 and 4-OH-E2 in normal human mammary epithelial MCF-10A cells was dose-dependent (Fig. 1 A,B). 4-OH-E2 induced significantly more ROS in these cells compared to E2 and 2-OHE2. The abilities of these estrogens to produce ROS were inhibited by overexpression of catalase or treatment with Ebselen or NAC (Fig. 1C). When MnSOD were over-expressed in these cells, ROS levels increased significantly compared to cells treated with estrogen alone (Fig. 1C). We also measured the cellular protease activities to rule out the possibility that the differential ROS levels were not as a result of differential cell densities or viability. Our results indicate that 4-OH-E2 is the most effective in generating intracellular ROS in MCF-10A cells. Mitochondria may be the major source of estrogen induced intracellular ROS, because overexpression of MnSOD, a mitochondria superoxide dismutase that converts superoxide to hydrogen peroxide, increased the ROS content maximally.

### Exposure of MCF-10A cells to 17β-estradiol (E2) and its catechol metabolites induced dose-dependent colony formation

**Figure 2:**
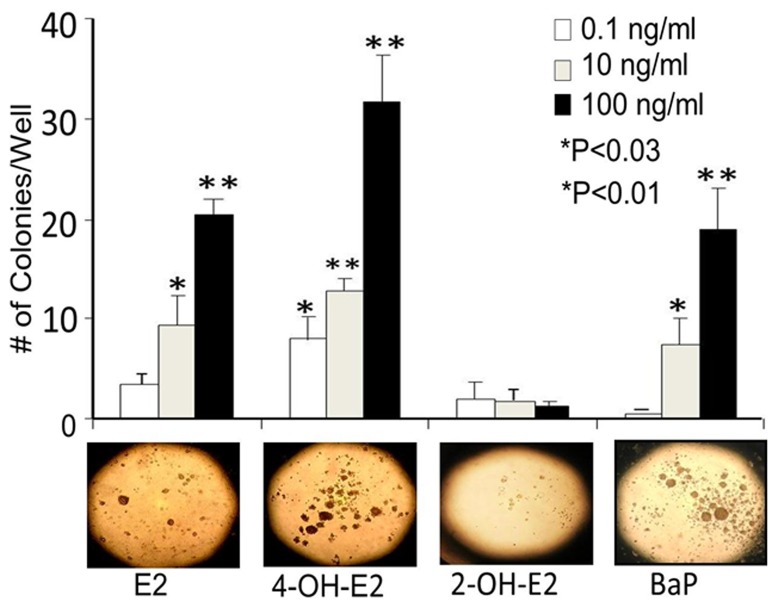
Exposure of MCF-10A cells to E2 and its hydroxy metabolites induced dose-dependent AIG positive colony formation (2A) and ROS modifiers inhibited estrogen-induced colony formation (2B). The cell transformation was carried out by a modified protocol of Russo's group (18). Briefly, MCF-10A cells were seeded at 30% density in a 10 cm dish. After 24 hrs of seeding, cells were exposed to E2 or its hydroxy metabolites. A treatment cycle includes a 48 hr starvation period, 48 hr treatment period (100 ng/ml of E2, 2-OH-E2, or 4-OH-E2), and 48 hr recovery period in DMEM-F12 media containing 10% horse serum (HS) and no growth supplements. Benzo(a)pyrene (BaP) was used as a positive control to show AIG positive colony formation. For inhibition of 4-OH-E2-induced cell transformation by ROS modifiers, MCF-10A cells were transfected with 50 MOI adenovirus expressing catalase or MnSOD or treated with an antioxidant Ebselen (40 uM). Cells overexpressing catalase or MnSOD or treated with Ebselen were exposed to a carcinogenic regimen of estrogen as described above. Anchorage independent growth, an indicator of neoplastic transformation of cells, was assessed in soft agar as previously described by Zhang et al (31) after 21 days. Images were acquired by using an Olympus C-5060 digital camera attached to the Nikon TE2000U inverted microscope with a 4x objective (bottom panel shows representative pictures of colonies in soft agar in both 2A and 2B). Colony efficiency was determined by a count of the number of colonies >63 um in diameter and data expressed as mean of five wells +/− S.D.

We used the anchorage independent growth (AIG) assay to examine cell transforming ability of E2 by detecting AIG positive colony formation. E2 exposure to ERα negative normal human breast epithelial MCF-10A cells produced dose dependent increase in the frequency of colony formation (Fig. 2 and Table 1). At 21 days, we did not detect any colony formation of cell masses of 100 micron or greater in vehicle (DMSO) treated wild type MCF-10A cells. The colonies detected in the first week of DMSO (vehicle) treated MCF-10A cells did not survive for 21 days. We found that repeated treatments of MCF-10A cells with various doses of E2 or its catechol metabolites induced *in vitro* transformation of MCF-10A cells in a dose dependent manner (Fig. 2 and Table 1). 4-OH-E2 is more potent in transforming normal mammary epithelial cells compared to E2, while 2-OHE2 is a weakly transforming metabolite of MCF-10A cells (Fig. 2 and Table 1).

**Table 1:** The frequency of in vitro cell transformation by estrogens.

Dosage (ng/ml)	E2	4-OH-E2	2-OH-E2
0	0	0	0
0.1	0.08±0.03	0.18±0.038	0.06±0.04
10	0.2±0.06	0.26±0.03	0.056±0.03
100	0.42±0.04	0.64±0.02	0.04±0.015

Anchorage independent growth, an indicator of neoplastic transformation of cells, was assessed as previously described by Zhang et al (31). Briefly, 5000 untreated and estrogen treated cells were seeded in each well and were grown for a minimum of 21 days over agar layer. The frequency of cell transformation was determined by counting the number of colonies that formed in the presence of E2, 4-OH-E2 or 2-OH-E2: (number of colonies formed/the total number of seeded cells) x 100.

### Clonogenic expansion and invasiveness of 4-OH-E2 transformed MCF-10A cells

**Figure 3:**
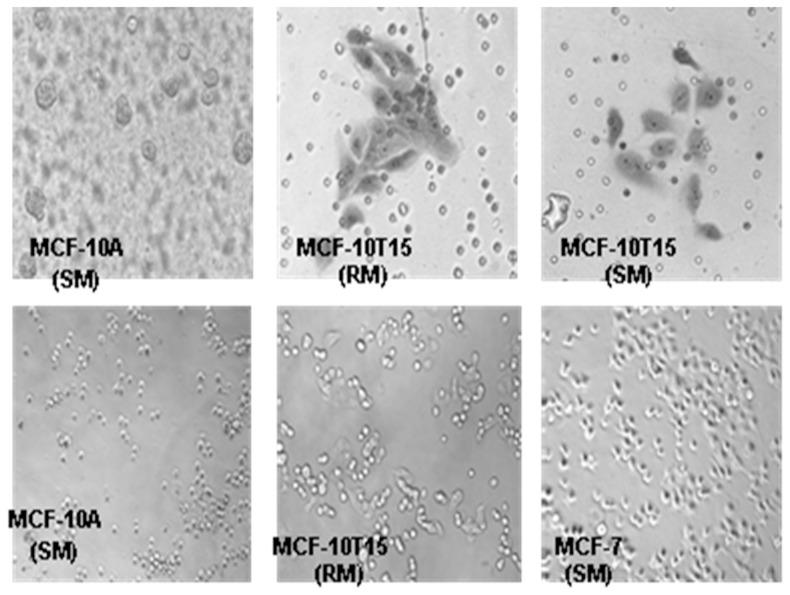
Clonogenic expansion and invasiveness of 4-OH-E2 transformed MCF-10A cells. Several 4-OH-E2 transformed MCF-10A colonies from each soft agar were picked up at the end of 21 days and were further cultured in media with 10% FBS (designated as a regular media -RM). Clones that survived up to the 21st passage were assessed for determining whether these cells have retained anchorage independent growth properties. Cells were fed twice per week and cultured for 21 days. Clones that were highly clonogenic (P21) were selected and the invasive property of this clone (MCF-10AT15) was analyzed by invasion assay in BD BioCoat™ Matrigel™ Invasion Chambers in the presence of either growth supplemented media ( SM) or media with only 10% FBS (RM) (Upper Panel). We also seeded these cells (Lower Panel) in a glass chamber because MCF-10A cells do not attach very well to glass in the first 16–24 hrs.

Since 4-OH-E2 induced the highest transforming frequency, we further examined whether 4-OH-E2 induced colonies are clonogenic. We picked several colonies from each soft agar at the end of 21 days and cultured them in media with 10% FBS (designated as a regular media -RM). Several of these clones did not survive beyond the 10th passage in RM. However, of the 5 that survived up to the 21st passage, one of the clones was highly clonogenic (P21) and responsive to E2. We labeled this clone as MCF-10T15. The invasive property of this clone (MCF-10AT15) was analyzed by invasion assay. We also seeded these cells in glass chamber as we previously found that MCF-10A cells don't attach very well to glass in the first 16–24 hrs. For the invasion assay, the chemotractant was either growth supplemented media (SM) or media with only 10% FBS (RM). Analysis of the invasive property of this clone MCF-10T15 by invasion assay showed that it is highly invasive (Fig. 3).

#### 3-D Spheroid formation of 4-OH-E2 transformed clone

**Figure 4:**
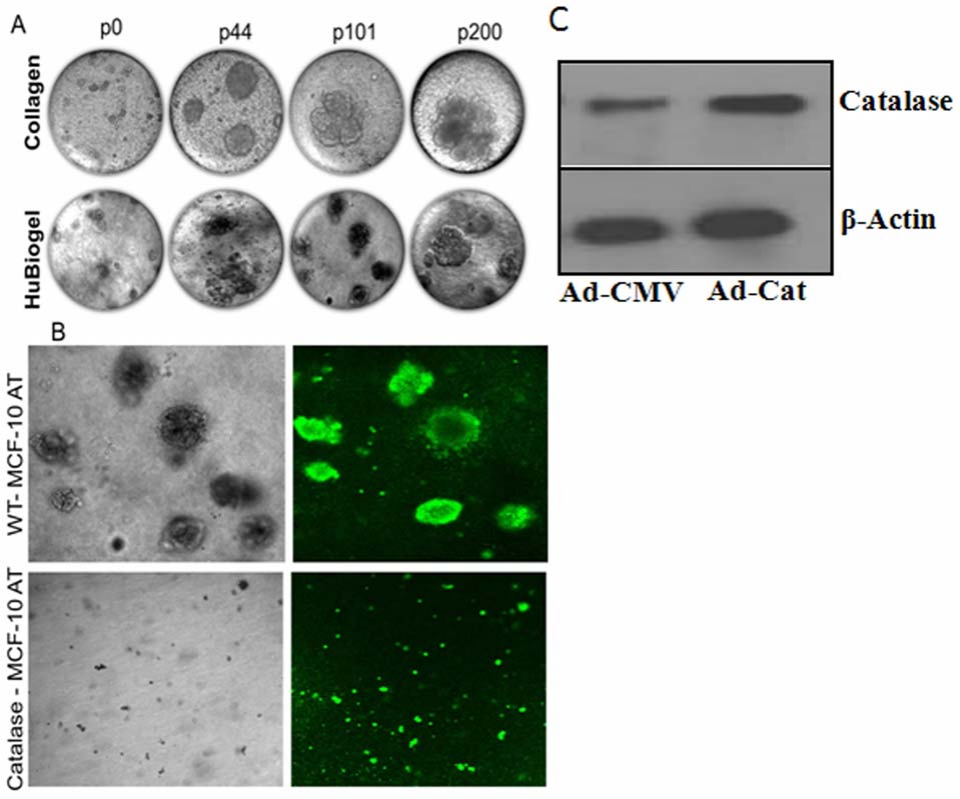
Spheriod Formation in Collagen and HuBiogel. **A.** Single colony from anchorage independent growth assay on agarose was aseptically picked at the end of 21^st^ day of culture. Clones were grown in growth factor reduced media (DMEM F12, 5% FCS, 4 ng/ml EGF, 2.0 ng/ml insulin, 100 ng/ml hydrocortisone, 1x penstrep) for 40–60 generations, then in growth factor depleted media (DMEM F12, 5% FCS, 1x penstrep) onward. Clones were assessed on collagen and HuBiogel for phenotypic evolutions. p0 indicates transformed cells prior to colony assay, p44-p200 are phenotypes of clone over several passages. Images were acquired with a hand held Nikon digital camera over an inverted microscope with 20x objective. **B.** MCF-10AT15 cells were infected with adenoviral vectors encoding catalase (Ad-catalase) or empty vector (Ad-CMV) as a control at 100 MOI in serum free media for 48 h. Wild type and catalase overexpressing MCF-10AT15 cells were mixed with 3D HuBiogelTM matrix containing DMEM-F12, seeded into a NASA engineered 55 ml rotating-wall vessel bioreactor systems, and incubated at 37°C for 16 days to further assess the colonogenic ability of 4-OH-E2 transformed MCF-10AT clone. Cell viability was checked with the Vibrant kit. All spheroids showing the green fluorophore indicated that cells in tumor spheroids were viable. **C.** Overexpression of catalase was confirmed by Western blot analysis using antibodies against catalase. β-Actin was processed in parallel as an internal control for protein loading.

To assess whether 4-OHE2 transformed MCF-10A cells are neoplastic, we picked a few colonies from the anchorage independent growth assay and cultured them repeatedly in growth factor reduced media, then assessed cells periodically for their ability to form spheroid structures in collagen coated 0.22 μm transwell inserts, or in a rotary vessel using HuBiogel, a mimetic of human stromal matrix. We found that over progressive passages, the clones in collagen matrix assumed a more heterogeneous population with small masses (p0) as opposed to homogenous population with aggressive phenotype (p200) (Fig. 4A).

The tumorigenic conversion ability of 4-OH-E2-transformed MCF-10A cells was further investigated by 3-D culture using HuBiogel^TM^. For 3-D culture, anchorage-independent MCF-10A human mammary gland epithelial cells transformed by 4-OH-E2 treatment were mixed with 3D HuBiogel^TM^ matrix containing DMEM-F12, seeded into 55 ml rotating-wall vessels and incubated at 37°C for 16 days. These conditions allow for the spontaneous formation of 3-D tissue-like spheroids of 4-OH-E2-transformed MCF-10A cells (Fig 4B). We found that overexpression of catalase inhibited tumor spheroid formation 4-OH-E2-transformed MCF-10A cells. Cells were labeled with CFSE using the Vybrant kit for checking viability. All spheroids showing the green fluorophore (Fig. 4B) indicate that cells in tumor spheroids are alive.

#### Loss of ductulogenicity in 4-OH-E2 transformed cells

**Figure 5:**
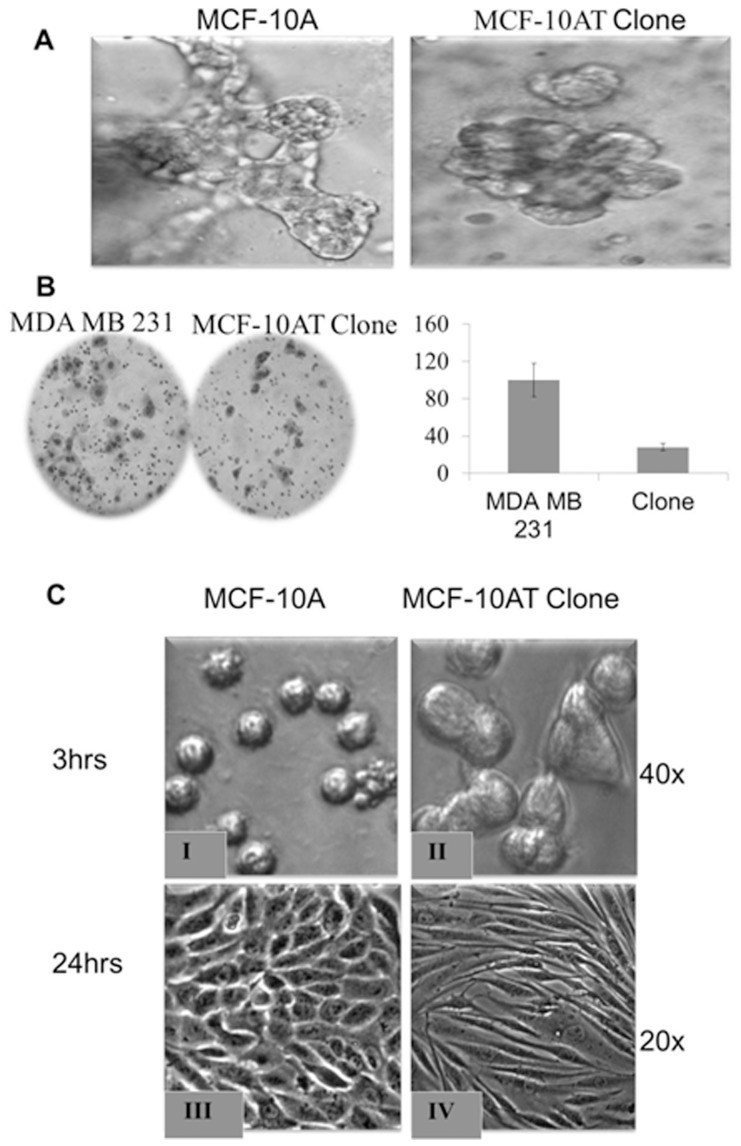
Phenotypic assessment of transformed cells. Clones from p21 cells were assessed for ductulogenesis (A), invasiveness (B) and seeding phenotypes (C). **A**) Ductulogenic assay was conducted on the collagen matrix. **B)** Invasive characteristic of clone was compared to MDA-MB 231 cells using invasion assay in BD BioCoat™ Matrigel™ Invasion Chambers. Histogram of invaded cells expressed as mean invaded cells per field. **C**) Phenotype of transformed clone (MCF-10AT) and wild type MCF-10A cells were captured through acquiring images of these cells after 3hrs and 24 hrs of seeding in a monlayer culture using an Olympus C-5060 digital camera attached to the Nikon TE2000U inverted microscope with 20x and 40x objectives.

The ability to form ductile structures is characteristic of normal mammary epithelial in a collagen matrix. Loss of this ability is a hallmark of transformed cells. Assessment of transformed cells in a collagen matrix indicates that these cells have indeed lost their ability to form ductile structures upon repeated treatment with 4-OHE2 (Fig. 5A). This phenotypic change was evident right from p0 cell population and continued even at p121.

### Aggressive phenotype enrichment

Initial invasion assessment of fifteen colonies from soft agar assay indicates that three colonies have acquired the ability to invade Matrigel matrix. These clones were subsequently cultured in growth factor reduced media over several generations and assessed periodically for invasive phenotype. We observed that successive passage of these clones increased their ability to invade Matrigel matrix. One of the clones termed clone c (MCF-10ATc) had actually acquired invasion capability that is about 30% that of MDA MB 231 cell line at passage (Fig. 5B). The other clones had lesser invasive abilities compared to MCF-10ATc (data not shown).

#### 4-OH-E2 treatment causes epithelial to non-epithelial transition in MCF10A cells

We found that the 4-OH-E2 transformed clones looked very different from WT both in morphology, size and time it takes to form a sheet upon seeding. Figure 5C shows that transformed clones were bigger than their WT counterpart in monolayer culture just after seeding (3 hrs). In addition, transformed cells displayed an abnormal differentiation pattern and loss of cell polarity, all phenotypes of cancer cells (Fig. 5C). The majority of clones exhibited morphological changes that resembled epithelial to non-epithelial transition. MCF10A cells showed highly organized cell-cell adhesion and cell contact, whereas 4-OH-E2 transformed-MCF10A cells had an elongated and refractive appearance with cell scattering and loss of cell-cell contacts. The cobblestone-like morphology of MCF10A cells at confluency was replaced in 4-OH-E2 transformed-MCF10A cells by a spindle-like fibroblastic morphology.

### Inhibition of 4-OH-E2-induced cell transformation by ROS modifiers

We used vehicle treated WT-MCF-10A cells as controls for all experiments, except adenovirus infection experiments. In adenovirus experiments, we used MCF-10A cells infected with empty adenovirus-CMV vector as controls. There was no difference in the number of colonies formed in WT-MCF-10A cells and MCF-10A cells infected with empty adenovirus-CMV vector as controls (background levels). Therefore, we have used the same controls as a representation for both adenovirus and non-adenovirus experiments.

First, we examined the effect of the pure antiestrogen ICI 182780 on cell transformation by treating MCF-10A cells with E2 or 4-OH-E2 (100 ng/ml) that produced the maximum-anchorage-independent growth in the presence of an equal concentration of ICI 182780. It has been shown previously that the pure antiestrogen ICI 182780 exerts dose-dependent growth inhibition on prostate cancer cells by an ER-beta-mediated pathway [34], [35]. The antiestrogen ICI 182780 did not prevent the anchorage independent growth of E2 or 4-OH-E2-treated MCF-10A cells (Table 2). This finding is in agreement with the report on normal ERα negative breast epithelial MCF-10F cells by Russo's research group showing that ICI 182780 does not abrogate E2 or 4-OH-E2-induced transformation of MCF-10F cells to neoplastic cells [19]. Our findings suggest that estrogen antagonist ICI-182-780 does not inhibit the in vitro transformation of MCF-10 A cell by E2 and 4-OH-E2.

**Table 2:** The estrogen antagonist ICI 182780 does not inhibit the in vitro transformation of MCF-10A cell by E2 and 4-OH-E2.

ICI 182780	E2 (100 ng/ml)	4-OH-E2 (100 ng/ml)
0	17.0±2.8	40.0±8.0
100 ng/ml	21.0±4.0	44.0±7.07

Anchorage independent growth of 5000 untreated and E2 or 4-OH-E2 (100 ng/ml) treated MCF-10A cells in the presence or absence of an equal concentration of ICI 182780 was monitored by growing these cells for a minimum of 21 days over agar layer. The number of colony formed in each well was determined by a count of the number of colonies >63αum in diameter and data expressed as mean of the number of colonies formed in the four wells +/− S.D.

**Figure 6:**
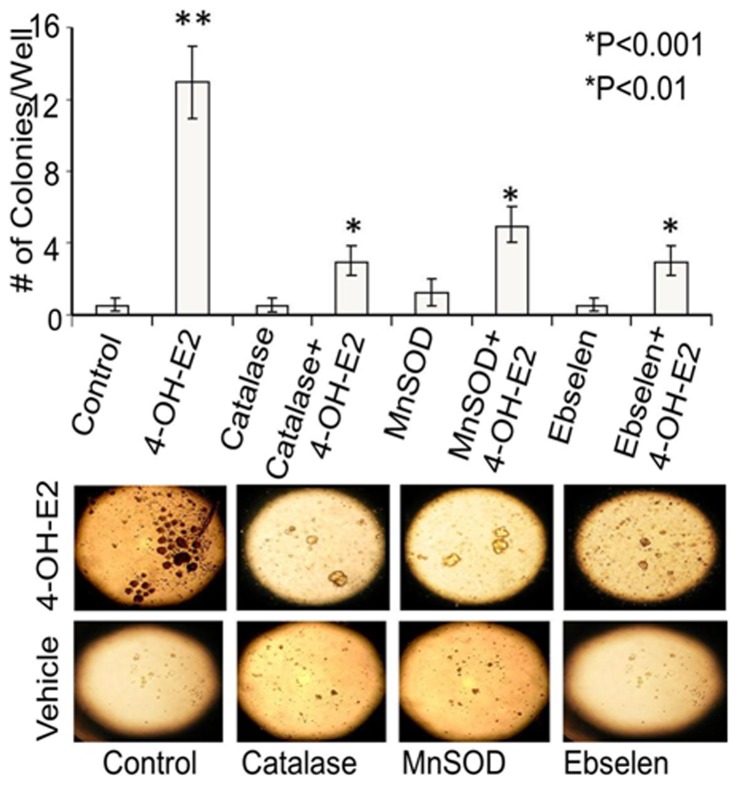
ROS modifiers inhibited estrogen-induced colony formation. For investigating inhibition of 4-OH-E2-induced cell transformation by ROS modifiers, MCF-10A cells were transfected with 100 MOI adenovirus expressing catalase or MnSOD or treated with an antioxidant Ebselen (40 uM). Cells overexpressing catalase or MnSOD or treated with Ebselen were exposed to a carcinogenic regimen of estrogen as described in the legend of figure 2. Anchorage independent growth was assessed in soft agar after 21 days. Images were acquired by using an Olympus C-5060 digital camera attached to the Nikon TE2000U inverted microscope with a 4x objective (bottom panel shows representative pictures of colonies in soft agar in both 2A and 2B). Colony efficiency was determined by a count of the number of colonies >63 um in diameter and data expressed as mean of five wells +/− S.D. *P<0.05, significantly different from 4-OH-E2 treatment. **P<0.05 indicates significantly different from control.

In cells overexpressed with adenovirus construct containing catalase and MnSOD that lowers oxidant production as well as in mtTFA silenced cells, E2 produced fewer colonies compared to E2 alone (Fig. 6). Treatment of cells with chemical ROS scavenger (Ebselen or NAC) significantly inhibited the abilities of E2 or 4-OHE2 to induce neoplastic transformation of MCF-10A cells as assessed by inability to form colonies and grow in soft agar assays (Fig. 6). This implies that oxidants induced by E2 and 4-OHE2 are necessary for tumorigenic transformation of MCF-10A cells and when oxidant levels were scavenged by biological and chemical antioxidants, estrogen induced transformation of mammary cells was inhibited.

**Figure 7:**
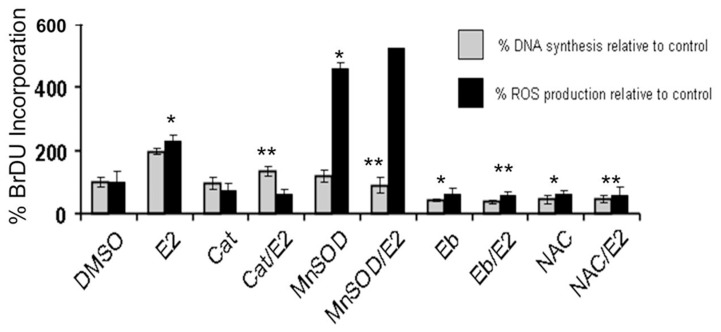
The growth of 4-OH-E2 transformed MCF-10AT clone was stimulated by 17 beta-estradiol (E2) and inhibited by both antioxidants, Ebselen and N-acetyl cysteine. Cells were grown in 96-well plates for 2 days in 10% FBS DMEM/F12 and serum starved 2 days prior to addition of E2 (100 pg/ml) for 18 h-48 h unless specified otherwise. Bromodeoxy uridine (BrdU) incorporation assay was used to measure DNA synthesis in transformed cells. Antioxidants ebselen, NAC, and catalase were pretreated for 2 h prior to the addition of E2. Colorimetric BrdUrd incorporation was measured at 450 nm with a plate reader. ROS production was measured by 10 uM H2DCFDA oxidation on a 96 well plate reader. Results are expressed as mean ± SD of three separate experiments with control set as 100%. *P<0.05, significantly different from control. **P<0.05 indicates significantly different from E2, (P<0.05).

The growth of the E2-induced transformed clone was highly responsive to E2 and was inhibited by Ebselen and N-acetyl cysteine. Antioxidants reduce E2-induced DNA synthesis and ROS formation in MCF-10AT transformed cells (Fig. 7). These cells respond to E2 in terms of producing ROS very similar to breast cancer cells.

### Inhibition of xenograft growth of adenocarcinoma in nude mice subcutaneously injected with 4-OH-E2 transformed breast epithelial cells by overexpression of catalase as well as co-treatment with Ebselen

**Figure 8:**
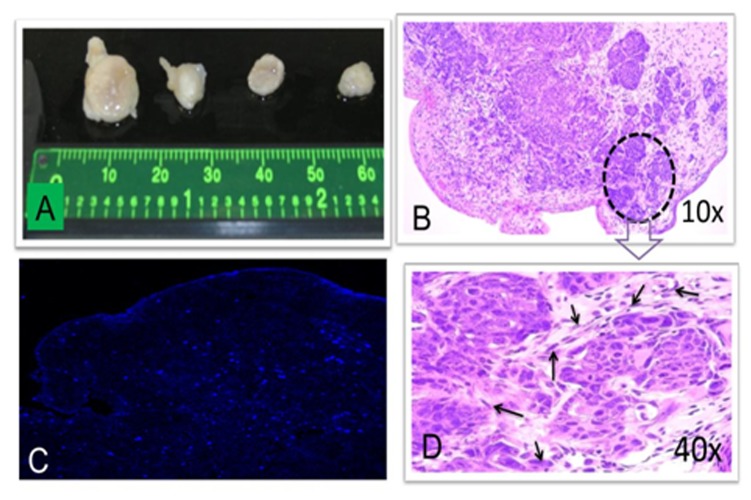
Inhibition of xenograft growth of malignant tumor in vivo by ROS modifiers. We selected 4-OH-E2 transformed AT cells thrice using the Matrigel invasion assay for aggressive phenotype enrichment. Cells that had invaded the matrix at the end of 72 hrs were cultured and re-verified by colony assay. These invasive cells were transfected with adenvovirus CMV vector or ad catalase or treated with 20 uM Ebselen. Mice were dorsally injected subcutaneously with 100 ul of Matrigel suspension of 5x105 WT, vector or catalase overexpressing MCF-10AT clone cells. Mice were injected daily with 17β-estradiol (0.125 mg/mouse). After 28 days, all mice were sacrificed, tumor from the injection site was dissected and tumors were weighed with portions of each fixed in neutral buffered formalin and embedded in paraffin for histological examination. A) Photograph of tumors from mice. Tumors from mouse, (B) Representative images of 10x of H&E stained section of tumor, (C) Representative images of 10x of Ki67 immuno-reactivity of tumor section detected by immunofluorescence confocal microscopy using Alexafluor 488, and (D) Representative images of 40x of H&E stained section of tumor.

Previous studies had failed to show xenograft growth of estrogen-transformed MCF-10A cells in mice [21], [22], [24]. We first selected highly invasive and migratory phenotypic enriched cells from 4-OH-E2 transformed cells using Matrigel invasion assay. For enrichment of highly invasive 4-OH-E2-transformed MCF-10AT cells, transformed cells that had invaded the matrix at the end of 72 hrs and formed a monolayer at the bottom well were cultured and re-verified on colony assay for anchorage independent growth. The cells from colonies were again subjected to migrate through the membrane and cultured and verified for colony formation. This process was repeated three times. Upon verification of colony growth, invasively enriched cells were suspended in 5 mg/ml Matrigel so that each injection of 100 μl contained 5×10^6^ cells. Cell suspensions were injected subcutaneously into 6 week old nude mice. In contrast to previous studies [21], [22], [24], invasively selected 4-OH-E2-transformed MCF10AT cells formed a palpable mass within 18–20 days (4/4 with Matrigel) (Fig. 8 and Table 3). After 28 days, xenografts injected with Matrigel had reached a mass of 260 mg. The average tumor weight at day 28 post injection was 260.0 mg/mice. However, the xenografts of MCF-10AT clone overexpressing catalase or co-treated with 20 uM Ebselen did not form palpable tumor. Similarly, normal MCF10A cells injected into nude mice were unable to form a palpable mass when injected with Matrigel (0/4). The xenografts with 4-OH-E2-transformed MCF10AT cell infected with empty adenovirus-CMV vector produced a palpable tumor of 210+/− 8.0 mg weight. Histologic analysis of the tumors by H&E staining revealed them to be poorly differentiated adenocarcinomas. The immunohistochemical assessment of the nuclear antigen Ki-67 showed the presence of Ki-67 positivity in tumors indicating that they are proliferative in nature. Taken together these data indicate that ROS modifiers, such as overexpression of catalase that converts hydrogen peroxide to water or a glutathione peroxidase mimic-Ebselen that modulates oxidative stress by enhancing thioredoxin reductase activity in 4-OH-E2-transformed MCF10AT cells is sufficient to inhibit xenograft growth of adenocarcinoma.

**Table 3:** Suppression of xenograft growth of 4-OH-E2 transformed MCF-10AT cells (i) by treatment with ROS scavengers – Ebselen and overexpression of antioxidant enzyme – catalase.

Experimental Groups	# of Mice	# of Mice with Tumors (%)	Tumor Weight (gm)	Duration (Days)
MCF-10A	4	0/4 (0%)	ND	28
MCF-10AT	4	4/4 (100%)	0.26±0.08	28
MCF-10AT/Vector	4	4/4 (100%)	0.21±0.04	28
MCF-10AT/Catalase^ovx^	4	0/4 (0%)	ND	28
MCF-10AT/Ebselen	4	0/4 (0%)	ND	28

ND  =  Not detected.

### 4-OHE2 induced ROS activates PI3K/KT signaling pathway

**Figure 9:**
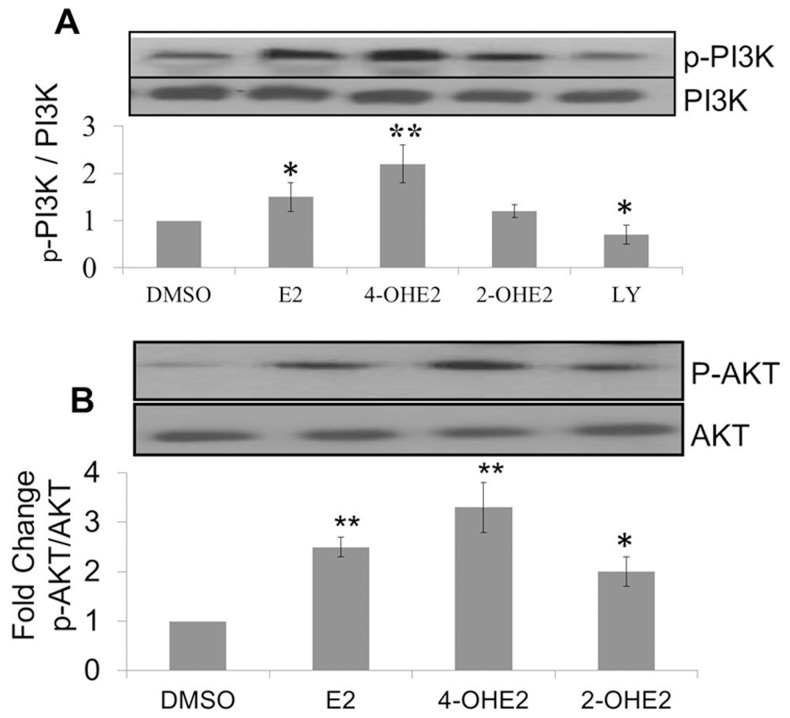
17 β-Estradiol (E2) and its metabolites differentially activate PI3K/AKT signaling pathway during mammary transformation. A. Estrogen-induced PI3K phosphotylation. B. Estrogen-induced AKT phosphotylation. MCF-10A cells were exposed to a carcinogenic regimen of E2 and its metabolites – 2-OH-E2 or 4-OH-E2 as described in the legend of figure 2. At the end of transformation process, cells were treated for additional 30 minutes with E2, 2-OH-E2 or 4-OH-E2, respectively. The cellular extracts from treated and controls cells were immuno-precipitated with PI3K or AKT specific monoclonal antibodies and followed by Western detection of PI3K or AKT phosphorylation using phosphor-threonine antibody. Results are expressed as mean fold change of three separate experiments. *P<0.05, significantly different from contol. **P<0.001 indicates significantly different from control.

**Figure 10:**
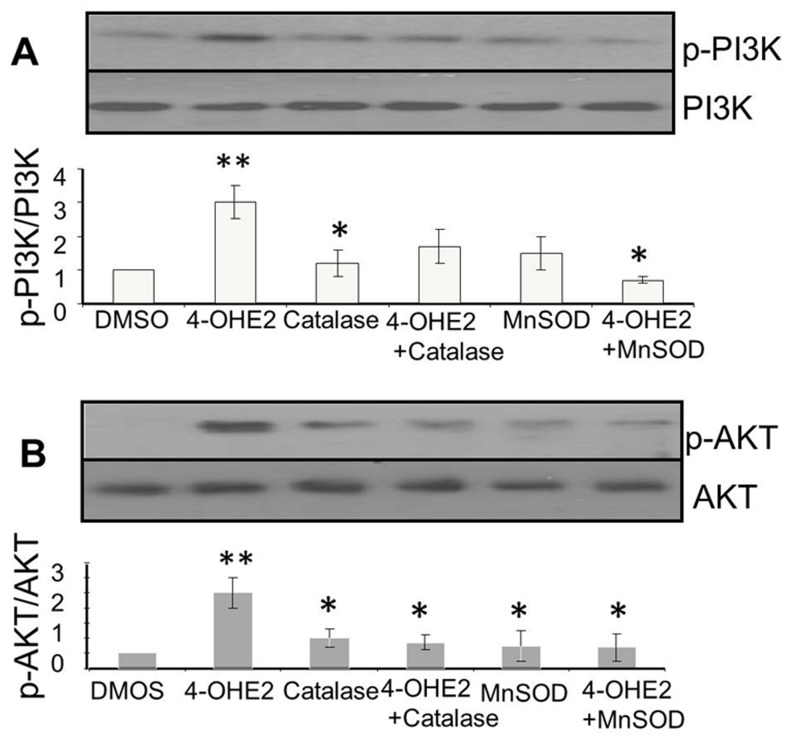
Inhibition of 4-OH-E2-induced phosphorylation of PI3K (A) and AKT (B) by biological ROS modifiers. A. Inhibition of 4-OH-E2-induced PI3K phosphotylation by overexpression of MnSOD and catalase. B. Inhibition of 4-OH-E2-induced AKT phosphotylation by overexpression of MnSOD and catalase. For investigating inhibition of 4-OH-E2-induced cell transformation by ROS modifiers, MCF-10A cells were transfected with 50 MOI adenovirus expressing catalase or MnSOD. Cells overexpressing catalase or MnSOD were exposed to a carcinogenic regimen of 4-OH-E2 as described in the legend of figure 2. At the end of transformation process, cells were treated for additional 30 minutes with 100 ng/ml 4-OHE2. The cellular extracts from treated and controls cells were immuno-precipitated with PI3K or AKT specific monoclonal antibodies and followed by Western detection of PI3K or AKT phosphorylation using phosphor-threonine antibody. Results are expressed as mean ± SD of three separate experiments with control set as 100%. *P<0.05 indicates significantly different from E2, (P<0.05). **P<0.05, significantly different from control.

**Figure 11:**
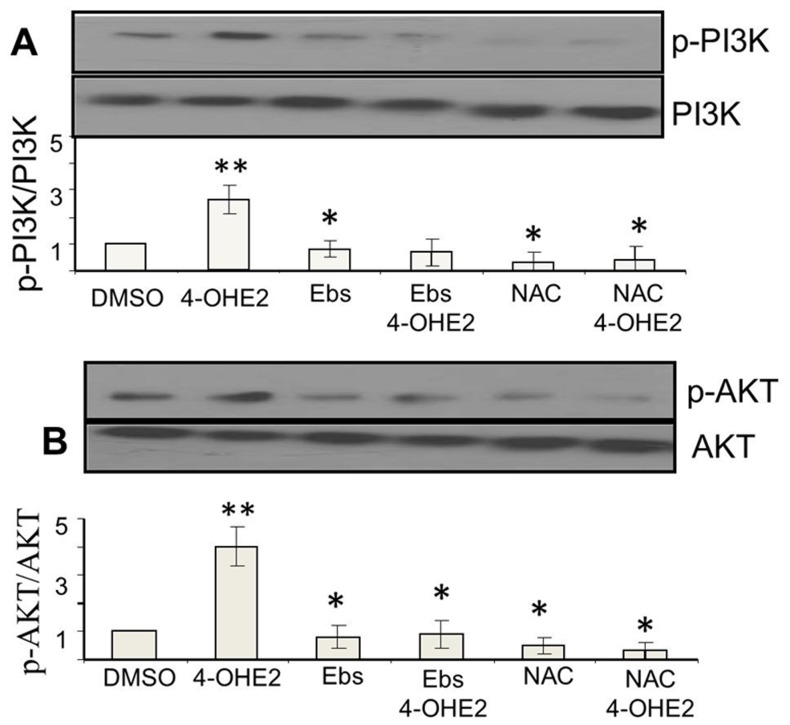
Inhibition of 4-OH-E2-induced phosphorylation of PI3K (A) and AKT (B) by Chemical ROS modifiers. A. Inhibition of 4-OH-E2-induced PI3K phosphotylation by co-treatment with Ebselen and N-acetylcysteine (NAC). B. Inhibition of 4-OH-E2-induced AKT phosphotylation by by co-treatment with Ebselen and NAC. For investigating inhibition of 4-OH-E2-induced cell transformation by ROS modifiers, MCF-10A cells pretreated for 2 hrs with 40 uM Ebselen or 10 mM NAC and were exposed to a carcinogenic regimen of 4-OH-E2 as described in the legend of figure 2. At the end of transformation process, cells were treated for additional 30 minutes with 100 ng/ml 4-OHE2. The cellular extracts from treated and controls cells were immuno-precipitated with PI3K or AKT specific monoclonal antibodies and followed by Western detection of PI3K or AKT phosphorylation using phosphor-threonine antibody. Results are expressed as mean ± SD of three separate experiments with control set as 100%. *P<0.05 indicates significantly different from E2, (P<0.05). **P<0.05, significantly different from control.

**Figure 12:**
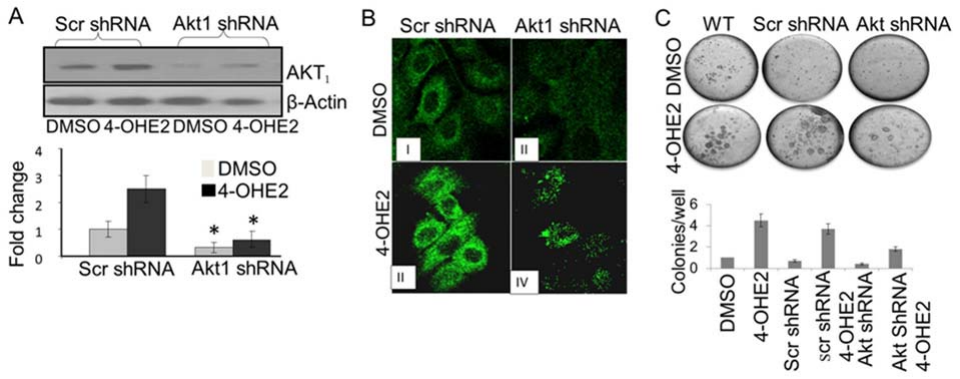
4-OH-E2-induced cell transformation is inhibited by AKT1 silencing. Inhibition of AKT expression by its silencing detected by Western Bloting (A) and confocal microscopy (B). (C) Detection of inhibition of 4-OH-E2-induced cell transformation by AKT1 silencing by anchorage-independent growth assay. The MCF-10A cells were transfected with pre-designed and verified human shRNA for AKT1 and control shRNA plasmid consisting of scrambled shRNA sequence that does not lead to the specific degradation of AKT1 (OriGene Technologies, Inc. Rockville, MD). These cells were exposed to a carcinogenic dose of 4-OH-E2 (10 ng/ml) as described in Fig. 2. The cellular extracts from treated and controls cells were separated on SDS-PAGE, transferred to the membrane, and followed by Western detection of AKT. Images of 40x of AKT immuno-reactivity of 4-OH-E2 treated wild type and Akt silenced MCF-10A cells were acquired by immunofluorescence confocal microscopy using Alexafluor 488. Anchorage independent growth, an indicator of neoplastic transformation of cells, was assessed in soft agar. Images were acquired by using an Olympus C-5060 digital camera attached to the Nikon TE2000U inverted microscope with a 4x objective. Colony efficiency was determined by a count of the number of colonies >63 um in diameter and data expressed as mean of five wells +/− S.D.

Consequences of elevated ROS in cells are apoptotic cell death, quiescence or cell transformation and neoplastic growth [36], [37] The signaling pathway associated with survival of cells under oxidative stress is attributed in part to activation of PI3K and AKT signaling pathways [37]–[39]. Therefore, we determined whether estrogen induced oxidants in normal mammary epithelial cells activate PI3K and AKT signaling pathways during neoplastic transformations of MCF-10A. We found that repeated treatments of MCF-10A cells with E2 and 4-OHE2 increased phosphorylation of both PI3K and AKT in cells treated with regimen of estrogen which produced cell transformation (Fig. 9). The activation of PI3K and AKT phosphorylation by 4-OHE2 were about 30% and 120% higher than E2 or 2-OHE2 respectively (Fig. 9). Phosphorylation of both PI3K and AKT was attenuated by co-treatment with either biological (Fig. 10) or chemical ROS modifiers (Fig. 11). Interestingly, we also observed that silencing of AKT_1_ (Fig 12A&B), significantly diminished 4-OHE2 induced neoplastic transformations of MCF-10A cells (Fig. 12). These data support that intracellular ROS induced by 4-OHE2 may activate AKT signaling pathway which favors survival and proliferation of cells, both required for malignant transformation.

### 4-OH-E2-induced ROS through redox signaling modulates cell cycle genes

**Figure 13:**
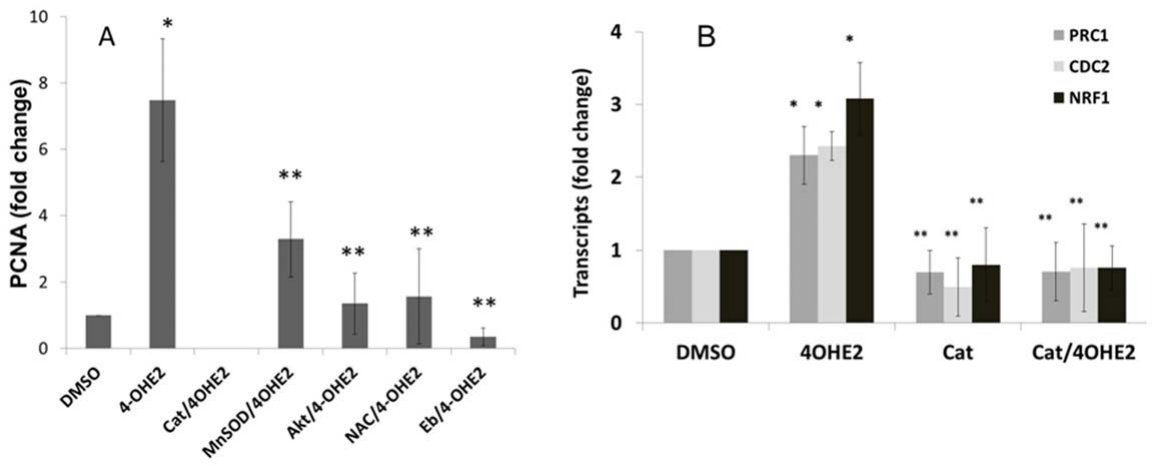
Up-regulation of cell cycle genes during 4-OHE_2_ induced neoplastic transformation of mammary cells and their expressions are inhibited by ROS modulators. A) Fold change of PCNA transcripts in 4-OHE2 transformed cells treated with ROS modulators, or transformed cells transfected with Akt1 RNAi. B) Fold change of cell cycle genes in 4-OHE2 transformed cells overexpressing catalase. MCF-10A cells were seeded for transformation as described in the legend of Fig. 2. At the end of transformation period, cells were treated for additional 18 hours with vehicles or 4-OH-E2 (100 ng/ml). For inhibition of 4-OH-E2-induced cell transformation by ROS modifiers, MCF-10A cells were transfected with 50 MOI adenovirus expressing catalase or MnSOD or treated with an antioxidant Ebselen (40 uM) or 10 mM NAC. Cells were first washed with cold PBS containing protease inhibitors, detached with trypsin, and RNA was isolated from 2.0×10^6^ cells. The TaqMan primers and probe recognizing PCNA, NRF1, PRC1, CDC2 and 18S were used in this study. Quantitative gene expression analysis was performed by TaqMan-based QRT−PCR on ABI 7700 (PE Applied Biosystems, Foster City, CA, USA). The fold change in gene expression was calculated using the {Delta} Ct method with 18S rRNA as the internal control. Results are expressed as mean ± SD of three separate experiments with control set as 100%. *P<0.05, significantly different from control. **P<0.05 indicates significantly different from 4-OH-E2, (P<0.05).

We investigated whether 4-OH-E2-induced ROS signaling is involved in the modulation of cell cycle genes during the conversion of normal breast epithelial cells to malignant cells. Using a normal cell line (MCF-10A) that develop transformed clones in response to 4-OH-E2, the expression of cell cycle genes, cdc2, PRC1 and PCNA and one of transcription factors that control the expression of these genes – nuclear respiratory factor-1 (NRF-1) was measured by real time RT-PCR [13]. After exposure of 8 h following two 48 h treatments with 4-OH-E2 (the treatment regimen that produces neoplastic cell transformation); we observed a significant increase in the mRNA expression of PRC1, Cdc2, NRF-1 and PCNA (Fig. 13). We then determined whether 4-OH-E2-induced expression of cell cycle genes was modulated by oxidants. MCF-10AT transformed cells were pretreated for 2 h with the antioxidants ebselen (20 μM) and NAC (10 mM) followed by a 8 h 4-OH-E2 treatment. As shown below in Fig. 13, real-time PCR analysis showed that overexpression of MnSOD and catalase as well as co-treatment with Ebselen and NAC markedly inhibited 4-OH-E2 induced PCNA expression (Fig. 13A) when compared to 4-OH-E2 treatment alone. Similarly, overexpression of catalase that detoxifies hydrogen peroxide significantly inhibited 4-OH-E2- induced expression of Cdc2, PRC1 and NRF1(Fig. 13B). Next, we evaluated whether the oxidant-dependent expression of cell cyclin genes was a function of AKT dependent signaling. We found that silencing of AKT1 significantly inhibited 4-OHE2 induced expression of the cell cycle gene, PCNA (Fig. 13A).

## Discussion

We present evidence here for the first time that reactive oxygen species (ROS) – induced by repeated exposures to 4-hydroxy-estradiol, a predominant catechol metabolite of 17 β-estradiol, caused malignant transformation of immortalized human mammary epithelial MCF-10A cells. 4-OH-E2 transformed cells are not only tumorigenic in mice but also display invasive properties as well as proliferation independent of growth factors. Co-treatments of 4-OH-E2 transformed cells with biological or chemical ROS scavengers prevented tumorigenic conversion of MCF-10A cells. This was further evident from inhibition of estrogen-induced breast tumor formation in the xenograft model by overexpression of the antioxidant enzyme, catalase or by co-treatment with a chemical antioxidant, Ebselen. These findings strongly support the idea that 4-OH-E2-induced ROS are required for estrogen-induced breast tumor formation. 4-OH-E2-induced malignant cell transformation may be mediated, in part, by redox-sensitive signal transduction pathways.

The mechanism by which estrogen is involved in the development of malignant breast lesions is not clear. Recent studies indicate that mammary tumors can develop in the absence of a functional ERα [25]. Although tamoxifen and other antiestrogens are thought to prevent cancer through their actions at the ER, other mechanisms cannot be ruled out as these compounds also block metabolism and redox cycling of estrogen and are free radical scavengers [26]. 4-OH-E2 induces an estrogenic response in the uterus of ERα null mice, and this response is not inhibited by the antiestrogen ICI182780 [27]. These findings suggest that estrogen-dependent growth of cells is regulated not only by nuclear ER-mediated genomic signaling pathways, but also by non-genomic pathway(s). Genomic and non-genomic actions of estrogen may produce complementary effects that are required for cellular transformation. Estrogen is genotoxic, as seen by the presence of DNA adducts in mammary tissues from ERKO/Wnt-1 mice [40], [41]. Although the formation of DNA adducts may lead to gene mutation, this type of DNA damage appears to be a late event arising from E2 metabolism. We and others have shown that mitochondria are significant targets of estrogen [28], [42]. Recently, we reported that physiological concentrations of E2 stimulate a rapid production of intracellular ROS in epithelial cells which depends on cell adhesion, the cytoskeleton, and integrins [28], [42]. These events occur earlier than ER-mediated genomic actions. E2-induced ROS production does not depend on the presence of ER on breast cancer cells as ER^-^cell lines MDA-MB 468 produced ROS equal to or more than that of ER^+^ MCF7, T47D, and ZR75cell lines [28].

4-OH-E2 has been implicated in transforming MCF-10A cells via ROS formation based on inhibition of anchorage independent growth of MCF-10A cells [24]. This study did not show *in vivo* tumor formation of transformed cells. The main difference between of our work and previous reports is that our transformed clones are tumorigenic in mice. The overexpression of catalase that converts hydrogen peroxide to water and Ebselen, a glutathione peroxidase mimic inhibited cell transformation and tumor formation. This is important because MCF-10A cells are easily transformed in an *in vitro* system, even by mild stress such as reduced growth factor media or hypoxic conditions [23]. Both E2 and 4-OH-E2 treatment of MCF-10A cells, increased the formation of ROS as compared to untreated cells, whereas 2-OHE2 induced the minimum increase in ROS formation in MCF-10A cells. Over-expression of biological ROS modifiers and chemical scavengers of ROS prevented 4-OH-E2-induced anchorage independent growth of MCF-10A cells. We observed similar results with 3-D culture of transformed cells using HuBiogel and xenograft tumor growth. These findings suggest that ROS induced by repeated exposures to 4-OH-E2, a predominant catechol metabolite of E2, cause transformation of immortalized human mammary epithelial cells with malignant growth in nude mice. Since 4-OH-E2 induces more ROS formation compared to E2 in MCF10A cells, the accumulation of 4-OH-E2 in the breast is expected to augment ROS formation here as well. 4-OH-E2 strongly binds to ER [43]–[46] and it takes longer to dissociate from the ER than E2 [45], [46]. The greater ROS production, ER action, and breast tissue accumulation of 4-OH-E2 compared to E2 may account for its greater carcinogenicity in MCF-10A cells.

It has been wrongly concluded by Parks et al [24] that redox cycling of catechol estrogen is the source of ROS. Catechol estrogens, particularly 4-OH-E2, via nonenzymatic auto-oxidation, may undergo redox cycling to produce reactive semiquinone and quinone intermediates with concomitant production of ROS [10]–[12]. However, this redox reaction of catechol estrogens is enhanced in the presence of Cu^2+^ or Fe^3+^ ions and by enzymatic catalysis by cytochrome P450 oxidases or peroxidases, which is accompanied with an increased generation of ROS. Furthermore, Parks et al [24] implied the contribution of redox cycling of catechol estrogen generating ROS based on indirect evidence using a non-specific inhibitor of cytochromes P450, SKF525A and dicumarol, an inhibitor of quinine reductase [47]. Dicumarol can also inhibit mitochondrial diaphorase, which is involved in reduction of Coenzyme Q10 in the mitochondria [48]. Similarly, SKF-525A inhibits mitochondrial oxidative metabolism in intact cells and isolated mitochondria [49]. Lower ROS formation observed in the presence of SKF525A and dicumarol may be as a result of inhibition of the mitochondrial electron transport chain. Increased ROS formation is observed within 30 seconds of E2 treatment [42]. Due to the speed of ROS production as observed in our study, it is unlikely that redox cycling of 4-OHE2 is the source of these oxidants. Furthermore, in our studies of E2-induced ROS generation in MCF-7 and other cells, hydroxylated estrogen metabolites or adducts immediately after addition of E2 were not detected which also rules out the possibility of ROS generation by redox cycling of hydroxylated estrogens.

Little is known about the potential direct involvement of estrogen-induced ROS in the development of breast cancer. We now know that the delicate intracellular interplay between oxidizing and reducing equivalents allows ROS to function as second messengers in signaling pathways controlling cellular proliferation and transformation [50], [51]. Recent studies implicate a role for ROS in cell transformation and several lines of indirect evidence support a role for ROS in the development of breast cancer [52], [53], We have previously reported that, in Syrian hamsters, estradiol-induced kidney tumor formation was reduced by the antioxidants N-acetylcysteine, vitamin C, sodium 2-mercaptoethanesulfonate (cytoprotective thiol-containing agent), and Ebselen (a substance with glutathione peroxidase-like activity) [54], [55]. Consistent with this finding, estrogen-induced testicular and uterine cancers are prevented by pentoxifylline, a compound with antioxidant effects stemming from its ability to block synthesis of the inflammatory mediators, IL-1β and TNFα [52]. Overexpression of manganese superoxide dismutase (MnSOD), the mitochondrial enzyme responsible for superoxide detoxification, blocks the appearance of malignant phenotypes [56], and the loss of this enzyme partly contributes to malignant phenotypes [57], [58]. Not surprisingly, MnSOD knockout mice exhibit increased oxidative DNA damage [59]. MnSOD expression is less frequently found in tumor cells of invasive breast carcinomas than in non-neoplastic breast epithelial cells [60]. Several epidemiological studies have shown that MnSOD polymorphic populations have an increased risk of breast cancer [61]–[63]. The recent findings that 4-OH-E2 accumulates in the breast tissue of cancer subjects [64]–[66] and predominant 4-hydroxylation of E2 occurs in the target organs of cancers [67]–[69] suggest that the target organ of cancer would be particularly sensitive to 4-OH-E2-induced ROS formation. In our studies, overexpression of catalase and antioxidant (Ebselen) prevented 4-OH-E2-induced anchorage independent growth of MCF-10A cells as well as xenograft tumor growth. These results provide support to the concept that that 4-OH-E2-induced ROS are required for estrogen-induced breast tumor formation.

How estrogen-induced ROS signaling is involved in breast carcinogenesis is not clear? While higher doses of ROS induce oxidative damage in the genome of cells leading to cell apoptosis, exposure of low levels of ROS produce genomic instability as well as transduce signals for cell growth, cell transformation and cell invasion. This view is consistent with our findings that estrogen-induced ROS can lead to increased phosphorylation of kinases, such as AKT. Several investigators have concluded that estrogen-induced AKT activation is promoted by membrane bound ERα or ERβ [70], [71]. There are no known functional motifs within the structure of the ER that can promote second messenger signaling. There are reports which show no correlation between ERα/β expression patterns and the activation of AKT-1/-2 in estrogen treated breast cancer cell lines. 17α-estradiol, through an ER independent mechanism, activates PI3K-AKT signaling [72]. Recently, Lee et.al. [70] reported that up-regulation of PI3K/AKT signaling by E2 is mediated through activation of ERα, but not ERβ. In ovarian cancer cells, 4-OH-E2 induces AKT phosphorylation while 2-OH-E2 did not [73]. Our study showed that 4-OH-E2 increased AKT phosphorylation in ERα lacking MCF-10 cells, while 2-OH-E2 did not increase AKT phosphorylation. The PI3K inhibitor, LY294002, and ROS modifiers blocked 4-OH-E2-induced AKT phosphorylation. AKT activity depends on its phosphorylation, which is positively regulated by PI3K and negatively regulated by a class of protein phosphatases (PPs) [74]. AKT can be activated by both E2 and H2O2 [70]–[76]. ROS reversibly regulate cysteine-based phosphatases [50]. The ability of 4-OH-E2 and H2O2 to activate AKT may be attributable to inactivation of cysteine-based phosphatases by ROS [50], [70]–[76]. The reversible inactivation of phosphatases, such CDC25A and PTEN, by estrogen-induced ROS may be a key component of AKT activation [50], [75]. Thus, some of the nongenomic pathways by which estrogen activates AKT pathways can be explained based on estrogen-induced ROS transducing signal to the respective specific phosphatse.

**Figure 14:**
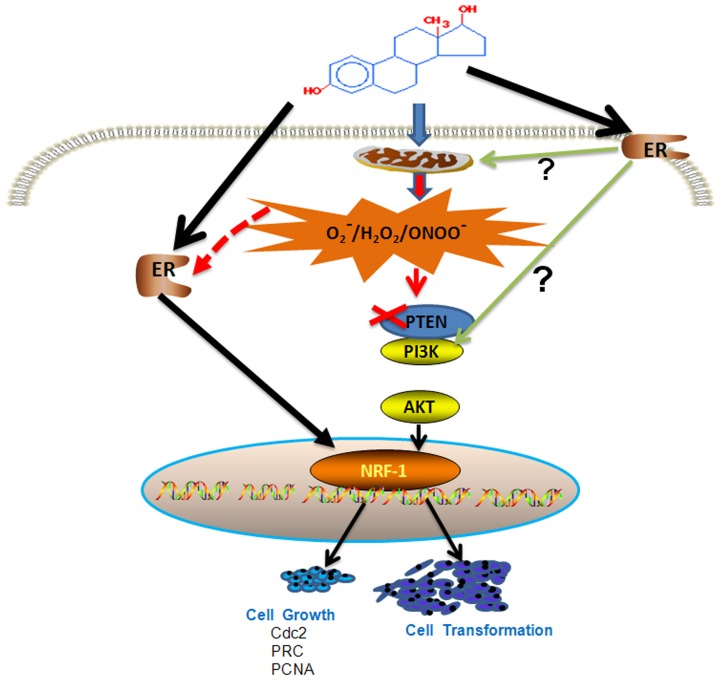
A scheme showing estrogen-induced ROS transduce signals to the nucleus for the activation of transcription factor NRF-1 to regulate their downstream target genes involved in cell transformation and cell cycle presumably through a redox-sensitive AKT pathway.

We and others have recently discovered that estrogen-induced oxidative bursts occur exclusively in perinuclear regions. This surge in ROS production may target inducible promoters, signaling transcription-initiation complex assembly and subsequently, driving estrogen-induced gene expression [29]. While studying mitochondrial regulators of cell cycle progression, we discovered that E2-induced G1 to S phase transition is associated with an increase in intracellular ROS levels [29]. These findings strongly support the idea that both E2-induced ROS and ER activity are required for breast cancer cell proliferation [29]. AKT by phosphorylating some of the transcription factors controls their transcriptional activity in a redox sensitive manner. The transcription factors AP-1, NRF-1, E2F, NFkB and CREB are responsive to both oxidants and estrogen. It is possible that estrogen-induced ROS transduce signals to the nucleus for the activation of transcription factors such as AP-1, CREB, E2F, NF-kB, and NRF-1 to regulate their downstream target genes involved in cell transformation, cell cycle, migration and invasion [29], [50]. In this study we found that the expression of cell cycle genes, cdc2, PRC1 and PCNA and one of transcription factors that control the expression of these genes – nuclear respiratory factor-1 (NRF-1) was significantly up-regulated during the 4-OH-E2-mediated malignant transformation process. The increased expression of these genes was inhibited by ROS modifiers as well as by silencing of AKT expression. Our findings suggest that 4-OH-E2-generated ROS activate AKT, which could then presumably directly phosphorylate and activate NRF-1 transcription factor controlling cell cycle, cell migration or cell invasion genes. Findings of this study provide support to the concept that up-regulation of NRF-1 mediated cell cycle genes through redox-sensitive AKT signal transduction pathway may contribute in 4-OH-E2-induced neoplastic growth of cells (Fig 14).

The PI3K/AKT signaling pathway seems ubiquitous to carcinogenic conversions [77]–[79]. Oxidant mediated hyperactivation of AKT can phosphorylate and inhibit pro-apoptotic proteins such as BAD and caspase 9 while phosphorylating and activating pro-growth transcription factors such as ASK1 and GSK3. The outcome of this hyperactivation could therefore be cells surviving and proliferating in a high oxidative state. If these cells have been initiated by acquisition of pre-tumorigenic lesions by 4-OHE2 metabolism, oxidant mediated growth of these cells could be the basis for malignant transformation of mammary cells. The loss of PTEN activity, hyperactivation of PI3K/AKT signaling pathway, excess estrogen exposure and oxidative stress have been implicated in breast carcinogenesis [50]. In this study, we also observed that PI3K/AKT signaling proteins were hyperactivated in MCF-10A cells treated repeatedly with estradiol and 4-OHE2 though the activations of 4-OHE2 were more than those of E2 and 2-OHE2 respectively. Importantly, chemical and biological antioxidant mitigated PI3K/AKT activations and inhibited estrogen induced mammary tumorigenesis. To rule out the possibility that antioxidant regulation of PI3K/AKT activations are not related to estrogen induced mammary tumorigenesis, we silenced AKT_1_ expression, the AKT isoform implicated in survival, growth and tumorigenesis of cells including mammary cells [77]–[79]. We found that silencing of this gene prevented estrogen's ability to transform MCF-10A cells. These data indicate that estrogen induced redox activation of PI3K/AKT signaling pathway is essential for mammary tumorigenesis.

A substantial number of experimental and epidemiological studies support an important role for AKT in tumorigenesis. PI3-kinase and AKT act as oncogenic determinants in several human cancers. AKT genes are amplified or overexpressed in gastric, ovarian, breast, pancreatic, and prostate cancers [80], [81]. AKT1 levels are higher in a panel of human breast carcinoma cell lines than in breast epithelial cells, particularly those with higher HER2 expression. AKT1 activity is increased by either E2 or IGF-I in estrogen-dependent MCF-7 cells, and both factors act synergistically to increase AKT1 activity and promote cell proliferation [82]. Transgenic mice expressing AIB1 (ER co-activator) in the mammary gland develop mammary hyperplasia and mammary carcinomas. Increased activation of the PI3K/AKT pathway is implicated in the development of mammary carcinoma in AIBI mice [83]. AKT activation amplifies the proliferation induced by cyclin D1 or HPV E7 during morphogenesis and cooperates with these oncoproteins to promote proliferation and morphogenesis in the absence of growth factors [82]. H-ras transformation of MCF-10A cells results in upregulation of MAP kinase and PI3-kinase signals [84], [85]. Similarly benzo(a)pyrene quinone is reported to induce anchorage-independent growth of MCF-10A cells which depends on the activation of PI3K/AKT activation [86]. Chronic activation of AKT2 leads to an increase of the events associated with tumorigenesis [87]. Most importantly, AKT activation disrupts mammary acinar architecture and enhances proliferation in an mTOR-dependent manner [83]. Our study showed that the exposure of 4-OH-E2 or E2 to normal human breast epithelial MCF-10A cells produced transformed phenotypes. These cells show increased AKT and increased cell number in the absence of EGF or insulin. The 4-OH-E2-induced expression of genes involved in proliferation was attenuated by the antioxidants. Overexpression of catalase and MnSOD also reduced the extent of 4-OH-E2-dependent anchorage-independent growth of MCF-10A cells and AKT activation. Taken together, these data indicate that 4-OH-E2-induced ROS activates the AKT pathway in MCF-10A cells and the generation 4-OH-E2-induced malignant phenotype of MCF-10A cell depends on the activation of PI3K/AKT pathway.

In summary, the major novel finding which emerged from this study is that ROS through the redox signaling pathway regulate estrogen-induced breast tumor formation. Estrogen-induced ROS can lead to increased phosphorylation of kinases, such as AKT, with this effect being attributed to the redox regulation of redox-sensitive phosphatse – PTEN. Inhibition of the increased expression of cell cycle genes, cdc2, PRC1 and PCNA and one of transcription factors that control the expression of these genes – nuclear respiratory factor-1 (NRF-1) by ROS modifiers as well as by silencing of AKT expression indicate that 4-OH-E2-induced cell transformation may be mediated, in part, by up-regulating NRF-1 mediated cell cycle genes through redox-sensitive AKT signal transduction pathway. Whether there is a convergence of both E_2_/ER mediated signaling (beta receptor and/or binding proteins) and E_2_ induced ROS signaling effects on the transcription factors which possibly act in synergy and contribute to the growth and cancer progression is under investigation in our laboratory. Findings of our study have major implications in understanding the role of estrogen in initiation and progression of breast cancer cells. Findings of this study not only provide a new paradigm in understanding the mechanism of estrogen-induced malignant cell transformation; it also provides important information for the design of new antioxidant-based drugs or new antioxidant gene therapy targeted for the prevention and treatment of estrogen-dependent breast cancer.
